# Exploration of the role of immune cells and cell therapy in hepatocellular carcinoma

**DOI:** 10.3389/fimmu.2025.1569150

**Published:** 2025-04-16

**Authors:** Tao Zhang, Cong Ren, Zhanyu Yang, Ning Zhang, Haowen Tang

**Affiliations:** ^1^ Faculty of Hepato-Pancreato-Biliary Surgery, Chinese People's Liberation Army (PLA) General Hospital, Beijing, China; ^2^ The Second Clinical College, Chongqing Medical University, Chongqing, China

**Keywords:** hepatocellular carcinoma, cell therapy, immune cells, immunotherapy, tumor microenvironment

## Abstract

Hepatocellular carcinoma stands as one of the foremost contributors to cancer-associated fatalities globally, and the limitations of traditional treatment methods have prompted researchers to explore new therapeutic options. Recently, cell therapy has emerged as a promising approach for HCC, showing significant potential in improving patient outcomes. This review article explores the use of cell therapy for HCC, covering different types, the mechanisms behind their effectiveness, recent advancements in clinical trials, and ongoing challenges. This article aims to provide insightful perspectives for future research and clinical applications in treating HCC by synthesizing current knowledge.

## Graphical Abstract

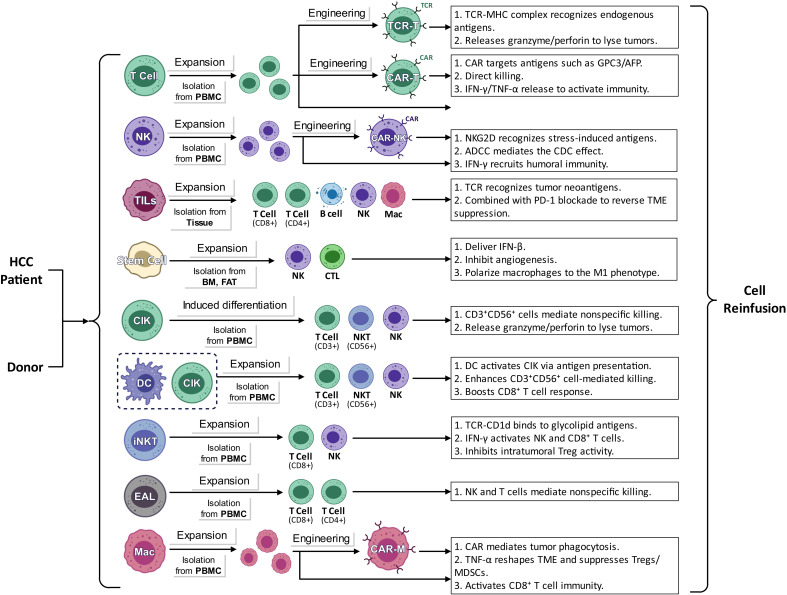

## 1 Introduction

Hepatocellular carcinoma (HCC) is a major global health issue, as it is the most prevalent form of primary liver cancer and a leading cause of cancer-related deaths around the world (1). The incidence of HCC varies by region, with China and East Africa reporting the highest rates, primarily due to widespread infections with hepatitis B virus (HBV) and hepatitis C virus (HCV) (2). Recently, the global burden of HCC has grown due to an increase in metabolic disorders. Among these metabolic disorders, metabolic dysfunction-associated steatotic liver disease (MASLD) is increasingly recognized as a significant risk factor for the development of HCC (3, 4). The pathogenesis of HCC is multifactorial, with chronic liver diseases, cirrhosis, and environmental factors such as aflatoxin exposure significantly contributing to its development (5–7).

Surgical approaches, including surgical resection, liver transplantation, and locoregional therapies, represent the only efficacious treatments for early-stage HCC. However, these treatments are only applicable to a small percentage of patients. For patients with advanced disease or underlying liver dysfunction, surgical options are often not viable treatment alternatives. Moreover, systemic therapies, such as chemotherapy, have primarily proven ineffective in treating HCC. HCC is characterized by its notable resistance to conventional chemotherapeutic agents (8). This underscores the urgent need for new treatment strategies to tackle the challenges of HCC. Based on in-depth research into the immune mechanisms of HCC, cell therapy has emerged as a promising alternative to treating this disease. Cell therapies use living cells to treat or prevent diseases and encompass various approaches, including stem cells, immune cells, and genetically modified cells (9).

Recent advancements in the field have highlighted the ability of specific therapies to more effectively target tumor cells and modify the tumor microenvironment, which can enhance the overall therapeutic response (10, 11). Immune cell-based therapies, particularly CAR T-cell therapy, have shown significant promise in treating hematological malignancies. They are currently being explored for their effectiveness against solid tumors, including hepatocellular carcinoma (12–19). This review aims to provide an overview of the latest insights into cellular therapies for managing HCC. By synthesizing recent findings and ongoing research efforts, we seek to clarify how cellular therapies could potentially revolutionize the treatment landscape for HCC and lead to improved patient outcomes. The following sections will examine various facets of cellular therapy, including its mechanisms of action and clinical implications in treating HCC.

## 2 Types and mechanisms of cell therapy in HCC

### 2.1 CAR-T cell therapy

Chimeric Antigen Receptor T cell(CAR-T) therapy is designed to quickly integrate specific chimeric antigen receptors into T lymphocytes, enhancing their ability to recognize and destroy cancer cells (20, 21). A chimeric antigen receptor typically consists of two main components: an antigen-binding domain derived from a monoclonal antibody targeting tumor-associated antigens and a signaling domain that triggers T-cell activation. This structure allows CAR-T cells to identify specific antigens found on tumor cells, resulting in their swift activation and proliferation, significantly boosting their effectiveness against tumors (22–24).

The effectiveness of CAR-T cell therapy relies on accurately targeting specific proteins highly expressed on tumor surfaces. Glypican 3 (GPC3) is one such protein found at elevated levels in more than 70% of hepatocellular carcinoma (HCC) cases, while it is nearly absent in normal tissues (25, 26). This heightened expression of GPC3 is also significantly linked to poorer prognoses for patients with hepatocellular carcinoma (27, 28). Studies using animal models have confirmed the efficacy of GPC3-CAR-T cells, with initial findings indicating that these cells have promising safety profiles and effectiveness in patients with GPC3-positive relapsed or refractory conditions (29–31). To improve the ability of CAR-T cells to infiltrate tumor environments, researchers have modified GPC3-CAR-T cells to express interleukin 7 (IL-7) and chemokine CCL19, resulting in positive outcomes in experimental studies (32). Another research on Interleukin-15-armored GPC3-CAR T cells for solid tumors, including liver cancer, showed that IL15 increases the expansion, intratumoral survival, and antitumor activity of GPC3-CAR-T cells in patients (33). Several studies have indicated that GPC3-targeted CAR-T cells that overexpress GLUT1 or AGK demonstrate improved CD8 T-cell persistence *in vivo* and greater antitumor effects in HCC (34). Additionally, GPC3-specific CAR-T cells engineered with IL-21 and CXCL9, combined with PD-1 blockade, have enhanced cytotoxic capabilities against hepatocellular carcinoma (35).

A significant number of liver cancer cases show increased levels of alpha-fetoprotein (AFP) in the blood, as this protein is released into the bloodstream. Consequently, several research teams have developed TCR-T cells designed to specifically target AFP (36, 37). Furthermore, early clinical trials have indicated that CAR-T cells targeting c-Met, NKG2D, CD133, and CEA have shown encouraging antitumor effects along with a satisfactory safety profile (12, 38–44).

CAR-T cells face two main challenges as they travel to and infiltrate tumor sites. The first challenge is the lack of essential chemokine receptors on T cells, which limits their ability to interact with chemokines released by tumor cells. This deficiency makes it difficult for CAR-T cells to reach the intended tumor location (45, 46). In the case of hepatocellular carcinoma, the situation is further complicated by a dense fibrotic matrix that reduces the expression of chemokines, significantly hindering the migration and infiltration of CAR-T cells into the tumor (47). Once these cells manage to enter the tumor, they encounter additional obstacles within the harsh tumor microenvironment (TME), which is marked by low oxygen levels and a lack of nutrients (48). Moreover, the TME in HCC is filled with various immunosuppressive cell types, such as regulatory T cells, tumor-associated macrophages, and fibroblasts. These cells can weaken the effectiveness of T-cell responses by releasing immunosuppressive factors and activating immune checkpoints (49).

Nonetheless, this therapeutic approach encounters several challenges, particularly notable adverse reactions like cytokine release syndrome (CRS), off-target toxicity, and neurotoxicity. These complications necessitate careful monitoring and management of patients (50, 51). Current research efforts are aimed at improving the durability and effectiveness of CAR-T cells while also expanding the therapeutic applications of CAR technology to include solid tumors, which have demonstrated higher resistance to this treatment strategy (52–55).

### 2.2 NK cell therapy

In the study of hepatocellular carcinoma, natural killer (NK) cells play a crucial role in suppressing tumors by effectively identifying and targeting cancerous cells. The interactions among these cells are complex and can vary significantly. Under normal physiological conditions, NK cells are highly capable of detecting and eliminating HCC cells (56). This recognition process involves various receptors, including NKG2D, NKp30, and NKp46, which bind to specific ligands found on tumor cells, such as MICA/B and ULBP (57). However, HCC tumor cells frequently downregulate the expression of these ligands as a strategy to evade immune detection, which diminishes the ability of NK cells to recognize and attack them (58). Research has demonstrated a direct correlation between the levels of NKG2D ligands on HCC cells and the cytotoxic activity of NK cells. Importantly, activating NKG2D has been shown to enhance the cytotoxic effects of NK cells against HCC (58). Furthermore, when NK cells are activated, they boost the overall immune response to tumors by producing cytokines like interferon-γ, which further supports anti-tumor activities (59).

The tumor microenvironment associated with hepatocellular carcinoma (HCC) plays a crucial role in affecting the functionality of natural killer (NK) cells, primarily through immunosuppressive mechanisms and metabolic dysregulation (60, 61). Low oxygen levels mark this microenvironment, the presence of immunosuppressive cells like regulatory T cells and hepatic stellate cells, and the secretion of tumor-associated cytokines such as interleukin-6 (IL-6) and transforming growth factor-β (TGF-β). Together, these factors lead to a reduction in NK cell activity (62, 63). For instance, elevated levels of IL-6 have been found to hinder NK cell function, resulting in decreased cytotoxic abilities and reduced cytokine production (62). Moreover, the secretion of soluble programmed death ligand-1 (sPD-L1) by HCC cells significantly contributes to the suppression of NK cell activity, causing NK cell exhaustion and a subsequent drop in their effectiveness (64, 65). Therefore, it is essential to either mitigate the immunosuppressive conditions within HCC or restore NK cell functionality to improve the outcomes of HCC therapies (59, 66).

Autologous natural killer cell therapy has significantly progressed in treating hepatocellular carcinoma (67). Numerous studies have shown that autologous natural killer cells can effectively recognize and eliminate tumor cells. However, the effectiveness of NK cells is often reduced in patients with HCC. Consequently, enhancing the activity of these cells has become a vital approach in HCC treatment. Recent clinical trials have demonstrated that expanding and activating autologous NK cells *in vitro*, especially when combined with cytokines like interleukin-15 (IL-15), significantly boosts their anti-tumor effectiveness (68). For instance, one study found that IL-2-activated autologous NK cells exhibited promising anti-tumor effects in a mouse model, which was also reflected in clinical responses from patients (69). Furthermore, research has highlighted a connection between NK cell functionality and the liver microenvironment, revealing that certain factors within this environment can promote NK cell growth and activation, thereby enhancing their ability to combat tumors (70).

Allogeneic natural killer cell therapy is emerging as a promising immunotherapeutic strategy, showing positive results in various clinical studies (71). Unlike autologous NK cells, which require time-consuming processes for cell expansion and activation within the patient, allogeneic NK cells can be obtained “off the shelf,” allowing for a quicker start to treatment. Research has shown that allogeneic NK cells sourced from healthy donors can effectively control the progression of hepatocellular carcinoma (HCC), with clinical trials reporting fewer side effects (69). For instance, a clinical trial involving patients with advanced HCC found that combining allogeneic NK cells with other immunotherapeutic approaches improved safety and increased effectiveness, leading to significantly better survival rates for patients (72). Additionally, there is ongoing research into chimeric antigen receptor-NK (CAR-NK) cells, with early results suggesting that these cells can specifically target tumor cells and enhance the anti-tumor response, opening up new possibilities for treating HCC (17, 22, 73, 74).

The tumor microenvironment poses significant challenges for therapies that utilize natural killer cells, creating hurdles for cancer immunotherapy. This environment is often marked by high levels of inhibitory cytokines, the presence of immunosuppressive cells, and hypoxic conditions, all of which severely limit the effectiveness and lifespan of NK cells. For instance, tumor cells can release inhibitory substances such as transforming growth factor beta (TGF-β) and interleukin-10 (IL-10), which hinder the activity and proliferation of NK cells (75). Additionally, the low oxygen levels typical of the tumor microenvironment negatively impact NK cell functions, making it difficult to accurately recognize and destroy tumor cells (76). In response to these challenges, recent advancements in NK cell-based therapies have led researchers to develop various combination treatment strategies specifically for hepatocellular carcinoma (HCC). One promising approach is the combination of NK cells with immune checkpoint inhibitors (ICIs), which have shown significant effectiveness in treating HCC. These immune checkpoint inhibitors, such as anti-PD-1/PD-L1 antibodies, enhance NK cells’ cytotoxic capabilities and cytokine production (76). Moreover, using these therapies together effectively counters the immune evasion strategies employed by liver cancer, promoting better tumor infiltration and activation of NK cells (59). The interaction between natural killer cells, targeted therapies, and the combination of NK cells with chemotherapy has garnered significant attention in treating hepatocellular carcinoma (HCC). Targeted therapeutic agents inhibit tumor cell growth directly and enhance anti-tumor responses by activating NK cell cytotoxicity (77). Additionally, these agents can help overcome drug resistance associated with targeted therapies (78). Chemotherapeutic drugs such as paclitaxel and cisplatin can increase the expression of ligands for NK cell-activating receptors on the surface of tumor cells. This elevation improves the ability of NK cells to recognize and eliminate tumor cells effectively (79, 80).

### 2.3 Tumor-infiltrating lymphocytes therapy

Tumor-infiltrating lymphocytes (TILs) are lymphocytes that infiltrate into tumor tissues, primarily T cells, B cells, natural killer cells, etc. These lymphocytes play a crucial role in immune surveillance and have anti-tumor effects within the tumor microenvironment (81, 82). They can recognize and attack cancerous cells, which has been linked to better overall survival rates (83, 84).

The process of isolating and expanding TILs is essential for their clinical application. Typically, TILs are collected from tumor tissue and then expanded *in vitro* (85). Modern techniques use specialized culture media and cytokines, particularly interleukin-2 (IL-2), to enhance TIL growth and improve functional abilities (86). Additionally, researchers are exploring more effective methods for cell separation, such as flow cytometry and magnetic bead sorting technology, to increase TIL’s purity and yield (87). These advancements improve the efficiency of TIL amplification and enhance their ability to kill tumor cells, laying a strong foundation for future immunotherapy applications. In the context of hepatocellular carcinoma (HCC), the TIL subpopulations most commonly studied include Foxp3+, CD8+, and CD4+ T cells, as well as B lymphocytes, NK cells, and macrophages, all of which are associated with prognostic outcomes. These advancements improve the efficiency of TIL amplification and enhance their ability to kill tumor cells, laying a strong foundation for future immunotherapy applications. In the context of hepatocellular carcinoma (HCC), the TIL subpopulations most commonly studied include Foxp3+, CD8+, and CD4+ T cells, as well as B lymphocytes, NK cells, and macrophages, all of which are associated with prognostic outcomes (88–91). Previous clinical trials have shown that administering TILs can significantly enhance patient survival rates after HCC hepatectomy (92, 93).

Future investigations should focus on integrating TILs with various immunotherapeutic strategies to improve treatment effectiveness. For instance, using anti-PD-1 monoclonal antibodies together with TILs has shown promising results (94). Studies suggest that agonists targeting co-stimulatory receptors, like GITR, may enhance the functionality of TILs. When these agents are used alongside PD-1 inhibitors, they could create a synergistic effect (95). TILs can serve as a standalone treatment option and work effectively in combination with other immunotherapeutic approaches, ultimately leading to better response rates and extended survival for patients with HCC.

Applying single-cell RNA sequencing technology offers a deep insight into the complex diversity found within tumor-infiltrating lymphocytes. This understanding allows for the precise tailoring of immunotherapy, ensuring that each patient receives a treatment plan that aligns with their specific immune profile (96). Furthermore, incorporating key biomarkers, such as PD-L1 expression levels and the extent of TIL infiltration, provides a dependable way to evaluate how well patients might respond to immunotherapy. This approach has opened doors to innovative personalized treatment strategies (97). By focusing on the unique characteristics of each patient, these tailored treatment plans not only improve the effectiveness of therapies but also minimize the risk of unnecessary side effects, significantly enhancing the overall quality of life for patients (98).

### 2.4 Stem cell therapy

Stem cell therapy for hepatocellular carcinoma focuses on three main aspects. First, it utilizes the regenerative capabilities of stem cells to help repair liver tissue. These stem cells can transform into hepatocytes, effectively replacing damaged cells and restoring liver function. Second, the therapy takes advantage of the immunomodulatory effects of stem cells to improve the tumor microenvironment. Stem cells can release various cytokines and bioactive molecules that influence the behavior of immune cells within the tumor area, reducing the immunosuppressive conditions and enhancing the body’s anti-tumor immune response. Finally, genetically modified stem cells are engineered to specifically target cancerous cells, which helps to limit tumor growth. These altered stem cells can be designed to recognize tumor cells, deliver anti-cancer agents, and induce programmed cell death in malignant cells, thereby preventing tumors’ growth and spread (99–102).

Numerous investigations have explored the therapeutic potential of mesenchymal stem cells (MSCs), liver cancer stem cells (LCSCs), and induced pluripotent stem cells (iPSCs) in treating hepatocellular carcinoma. MSCs are recognized for their significant capabilities in tissue repair, and research indicates that they can enhance HCC outcomes by reducing tumor-related inflammation and promoting liver regeneration (103). For instance, in an animal study, administering MSCs derived from bone marrow led to decreased levels of pro-inflammatory cytokines, such as tumor necrosis factor-alpha (TNF-α) and interleukins (IL-2, IL-10), which markedly improved liver function in rats modeling HCC and facilitated liver regeneration (104). Furthermore, MSCs have been shown to reduce tumor-associated immunosuppression by inhibiting T cells’ activation and proliferation while promoting the generation of regulatory T cells (Tregs), thereby enhancing immune tolerance and inhibiting tumor progression (103). These immunomodulatory properties of MSCs make stem cell therapy a promising strategy for HCC treatment. On the other hand, cancer stem cells (CSCs) in liver cancer possess a strong capacity for self-renewal, diverse differentiation potential, and the ability to initiate tumors. These cells play a crucial role in tumor progression, metastasis, and drug resistance, making them pivotal in the recurrence and metastasis of HCC (105). Studies have shown that a combination of 5-fluorouracil and a CD13 inhibitor can effectively suppress the proliferation of LCSCs and reduce tumor burden (106).

Induced pluripotent stem cells (iPSCs) are created by reprogramming somatic cells to express specific transcription factors, resulting in cells that can differentiate into any cell type, similar to embryonic stem cells. One of the main advantages of iPSCs is that they can be derived from various cell types, which minimizes ethical concerns. This feature allows researchers to isolate iPSCs from cells obtained from patients, paving the way for personalized treatment options. In the study of hepatocellular carcinoma (HCC), iPSCs are particularly useful as they provide models to explore the mechanisms of cancer development and to evaluate how liver cancer responds to different drugs (103). Furthermore, researchers can guide iPSCs to differentiate into hepatocyte-like cells using specific induction techniques, offering new strategies for treating HCC (107). Additionally, adipose-derived stem cells (ADSCs) hold considerable promise for HCC treatment, as they can promote liver tissue regeneration and repair by releasing various bioactive factors (103).

### 2.5 TCR-T cell therapy

Engineered T cell therapy, specifically T cell receptor (TCR) therapy, is an innovative approach in cellular immunotherapy designed to reprogram patients’ T cells to target and destroy tumor cells. This strategy relies on the ability of TCRs to recognize tumor-specific antigens presented by major histocompatibility complex (MHC) molecules, which activate the T cell-mediated immune response against tumors. TCR-T cells can identify unique antigens found on the surfaces of tumor cells, triggering a cytotoxic response that leads to eliminating these cancerous cells. Research has shown that TCR-T cell therapy is promising in clinical applications for various solid tumors, particularly highlighting its potential effectiveness in treating resistant tumors like hepatocellular carcinoma (108, 109).

Recent advancements have been made in studying hepatitis B virus (HBV)-specific T cell receptor redirected T (HBV-TCR-T) cells, particularly in HBV-associated hepatocellular carcinoma. In a clinical trial, one participant who received HBV-TCR-T cell therapy achieved a partial remission of 27.7 months. Moreover, most patients showed a significant decrease in both HBsAg and HBV DNA levels following treatment, highlighting the targeted effectiveness of this therapeutic strategy (110). Another clinical study, identified by clinical trial number NCT05339321, demonstrated the ability of genetically modified T cells to specifically target hepatocytes that express hepatitis B surface antigen and those involved in hepatocellular carcinoma (111). Additionally, a different clinical investigation (NCT02719782) emphasized the safety and anti-tumor efficacy of mRNA electroporated HBV-specific TCR-T cells (112). A significant challenge related to this therapeutic approach is the limited specificity that comes with T cell receptor (TCR) recognition. Most TCRs are designed to target specific antigenic epitopes, which limits their ability to recognize a broader range of targets. For example, TCRs developed to target hepatocellular carcinoma may not effectively recognize all tumor cells, especially when there are variations in the antigens present on the tumor cells.

Therapeutic strategies that combine different treatment modalities, especially those involving T-cell receptor (TCR)-engineered T cells, show significant promise in managing hepatocellular carcinoma (HCC). Integrating cytokines like interleukin-21 (IL-21) with TCR-T cell therapy can significantly enhance anti-tumor responses. IL-21 promotes the growth of TCR-T cells and supports their development into memory T cells, which boosts their effectiveness against tumors. Furthermore, IL-21 is crucial in reducing the expression of programmed cell death protein 1 (PD-1), which helps decrease cell death and strengthens the anti-tumor capabilities of TCR-T cells (113). Additionally, combining TCR-T cells with small molecule agents, such as Atovaquone, has improved treatment outcomes. Atovaquone increases the cytotoxic effects of TCR-T cells by triggering ferroptosis, which further hinders the progression of HCC (114). By enhancing the effectiveness of each therapeutic approach and reducing the chances of resistance, these combination therapy strategies pave the way for a more personalized and targeted treatment approach for HCC.

CRISPR/Cas9 technology for editing T-cell receptors (TCRs) allows for more precisely introducing these receptors into T cells. This accuracy ensures that TCRs are consistently expressed in T lymphocytes, which enhances their ability to fight tumors in living organisms (115). Simultaneously, the development of TCR-engineered T cells aimed at specific antigens, like glypican-3 (GPC3) and alpha-fetoprotein (AFP), has demonstrated promising safety and effectiveness in clinical trials (116).

### 2.6 CIK cell therapy

Cytokine-induced killer (CIK) cells represent an ex vivo-expanded heterogeneous immunocyte subset, mainly consisting of CD3+ and CD56+ T lymphocytes with dual T-cell and natural killer (NK)—like phenotypic characteristics (117). The expansion of CIK cells in the lab involves a specific sequence of cytokine treatments. First, interferon-γ (IFN-γ) is used to activate antigen-presenting cells. After this initial step, anti-CD3 monoclonal antibodies and interleukin-2 (IL-2) are introduced at specific times to promote the growth and development of these cells. The combined effects of these cytokines allow CIK cells to participate in adaptive and innate immune responses through two main ways of attacking. The first way involves recognizing antigens presented by major histocompatibility complex (MHC) molecules through T-cell receptors (TCRs). The second way allows CIK cells to use a receptor called NKG2D to detect stress-induced ligands like MIC-A/B and ULBP1-4, which means they can act without relying on MHC. This approach combines the activation of cytokines (IL-2 and IFN-γ) with the stimulation of anti-CD3 antibodies to create effector cells that are highly effective in killing cancer cells through perforin/granzyme pathways and producing Th1-type cytokines. As a result, CIK cells show enhanced abilities to recognize tumors compared to traditional lymphocyte therapies (118, 119).

CIK cells have become a significant focus in the field of immunotherapy for hepatocellular carcinoma. Clinical trials have highlighted the potential of CIK cells to enhance anti-tumor responses in patients with HCC. Clinical trials have highlighted the potential of CIK cells to enhance anti-tumor responses in patients with HCC. For example, a notable study involving 264 participants found that patients receiving CIK cell therapy, whether as a standalone treatment or alongside surgical procedures or transcatheter arterial chemoembolization (TACE), showed significantly improved overall survival (OS) compared to those undergoing standard therapies alone. The Kaplan-Meier analysis further revealed that patients who received both surgery and CIK therapy had better OS rates than those who only had surgery, with a statistically significant difference (P < 0.001). Additionally, incorporating CIK therapy into TACE regimens enhanced both OS and progression-free survival (PFS) (120). Another study demonstrated that CIK cells derived from HCC patients exhibited substantial cytotoxicity against various tumor cell lines, highlighting their effectiveness in targeting HCC cells (121). A comprehensive meta-analysis that combined results from multiple studies confirmed that CIK therapy significantly improves OS and reduces recurrence rates among HCC patients, reinforcing the therapeutic potential of CIK cells in clinical settings (122). Furthermore, the effectiveness of CIK therapy is linked to its ability to trigger a robust immune response. For instance, one study showed that CIK cells have high levels of activating receptors and low levels of immune checkpoint molecules, indicating their readiness to engage in anti-tumor activities (123).

The combination of cytokine-induced killer cell therapy with immune checkpoint inhibitors has shown promising results in treating hepatocellular carcinoma. Studies suggest that some patients who received CIK therapy before anti-PD-1 antibody treatment experienced complete responses, indicating a potential synergistic effect between these two treatment strategies (124). This synergy is thought to arise from the ability of CIK cells to enhance tumor-specific immune responses. In contrast, immune checkpoint inhibitors work to reduce the inhibitory signals that dampen T-cell activity in the tumor microenvironment. Moreover, a new strategy involves administering CIK cells with dendritic cells (DCs), leading to a DC-CIK combination therapy. This approach has been associated with increased immune activation and better anti-tumor responses in various cancers, including HCC (125, 126). In this context, DCs serve as powerful antigen-presenting cells that can further stimulate the growth and activation of CIK cells, thereby boosting their ability to effectively target and destroy HCC cells.

Novel methodologies, such as gas-permeable culture systems, have significantly enhanced the efficiency of cytokine-induced killer cell expansion while preserving their functional characteristics. A comparative analysis between traditional culture techniques and gas-permeable systems showed that CIK cells grown in these advanced systems exhibited improved proliferation rates and retained their ability to target myeloid leukemia cell lines effectively. This finding underscores these cells’ potential for large-scale clinical production (127). Incorporating various cytokines and growth factors into the culture media has also been explored to enhance the functional properties of CIK cells. For instance, adding N-acetylcysteine (NAC) during the culture process has been shown to significantly increase the cytotoxicity of CIK cells against cancer cell lines by promoting their proliferation and enhancing cytokine production (128).

### 2.7 DC-CIK cell therapy

DC-CIK cell therapy, which combines dendritic cells (DCs) with cytokine-induced killer (CIK) cells, has emerged as a promising immunotherapeutic strategy for hepatocellular carcinoma (HCC), supported by an increasing amount of clinical evidence demonstrating its effectiveness in improving patient survival and overall quality of life. A meta-analysis involving 3,756 HCC patients revealed that adding DC-CIK therapy to standard treatment methods, such as surgical resection or locoregional therapies, significantly enhances overall survival (OS) rates compared to conventional treatment alone (129). Notably, DC-CIK therapy has been found to substantially reduce recurrence rates, especially in patients who have undergone curative procedures like hepatectomy or liver transplantation. Clinical trials have shown a 32% reduction in early postoperative recurrence (within two years) when DC-CIK therapy is used as an adjunctive treatment (130). Additionally, recent multicenter studies in Eastern China have reinforced these findings, highlighting the increased antitumor effectiveness of DC-CIK therapy when combined with multimodal approaches such as microwave ablation, chemotherapy, or transarterial chemoembolization (TACE). For instance, a phase II clinical trial indicated that the combination of DC-CIK and TACE led to a median progression-free survival (PFS) of 14.6 months, compared to 9.8 months for TACE alone (HR=0.62; P<0.01) (131). Mechanistically, DC-CIK therapy improves the presentation of tumor antigens by DCs while simultaneously activating CIK cell-mediated cytotoxicity against HCC cells that lack MHC class I, thus overcoming significant challenges associated with traditional immunotherapy methods.

The combination of dendritic cell-cytokine-induced killer (DC-CIK) cell therapy with targeted agents has shown impressive synergistic effects in treating hepatocellular carcinoma (HCC). Research indicates that immune checkpoint inhibitors, especially those targeting PD-1 like pembrolizumab, significantly boost the cytotoxic effectiveness of DC-CIK cells by blocking the PD-1/PD-L1 interaction. This blockage helps reverse T-cell exhaustion and enhances the tumor-killing activity of DC-CIK cells, which is linked to better patient survival rates (132). Moreover, immunohistochemical studies have demonstrated increased CD8+ T-cell infiltration in tumors following combination therapy, indicating a heightened immune response (129). Simultaneously using DC-CIK and targeted therapies positively impacts the tumor microenvironment (TME). Targeted therapies can modify the TME, creating a more supportive environment for immune cell infiltration and function, thereby increasing the therapeutic effects of DC-CIK cells. Evidence shows that targeted therapies significantly improve the growth and cytotoxic abilities of DC-CIK cells in patients (130). A systematic review and meta-analysis have highlighted that DC-CIK immunotherapy has considerable potential for enhancing survival and response rates in cases of solid tumors (133).

### 2.8 iNKT cell therapy

Natural killer T (NKT) cells represent a unique subset of T lymphocytes characterized by co-expression of T-cell receptors and natural killer cell markers (134). Although they originate from the T-cell lineage, NKT cells exhibit both morphological and functional similarities to NK cells. They play a crucial role in bridging innate and adaptive immune responses by quickly releasing cytokines, essential during the early stages of immune reactions (135).

The invariant NKT (iNKT) cell subset, a predominant subtype of NKT cells, develops from CD4+ and CD8+ double-positive thymocytes (136). These cells are notable for their invariant TCR chain, which explicitly recognizes lipid antigens presented by CD1d molecules on antigen-presenting cells (APCs) (137). Upon activation by α-galactosylceramide (α-GalCer), a synthetic glycolipid antigen loaded onto CD1d, iNKT cells exhibit dual effector functions: 1) rapid secretion of both Th1-type (e.g., IFN-γ, TNF-α) and Th2-type (e.g., IL-4, IL-13) cytokines (138); 2) direct cytotoxic activity against tumor cells via perforin/granzyme pathways (139). Furthermore, activated iNKT cells enhance antitumor immunity by facilitating interactions with NK cells and cytotoxic T lymphocytes (CTLs) through CD40L-CD40 signaling and cytokine networks, thereby strengthening immune responses across various compartments (140).

Numerous clinical investigations are currently underway to evaluate the safety and effectiveness of various treatments for hepatocellular carcinoma (HCC). One notable study is a phase I clinical trial (NCT03175679) conducted at Beijing YouAn Hospital, which enrolled ten patients diagnosed with HCC at stages B/C according to the Barcelona Clinic Liver Cancer classification. In this trial, researchers isolated autologous invariant natural killer T (iNKT) cells from the patient’s peripheral blood mononuclear cells, expanded them, and pulsed them with α-GalCer before infusion. The trial results indicated that the administration of expanded iNKT cells was safe and well-tolerated, with most treatment-related adverse events classified as grade 1-2. Preliminary findings suggested that the infused iNKT cells triggered significant T-helper 1-like immune responses, potentially contributing to antitumor activity. Additionally, assessments conducted after infusion showed increased levels of circulating iNKT cells and activated natural killer (NK) cells among the patients, indicating a likely enhancement of antitumor immune responses (141). Furthermore, a phase II randomized controlled trial explored the effects of iNKT cell infusion combined with transcatheter arterial embolization (TAE) in patients with unresectable HCC who had previously failed TACE. This combined approach significantly improved progression-free survival (PFS) compared to TAE alone, highlighting the promising role of iNKT cell therapy in managing HCC (142). These findings are further supported by additional studies that underscore the antitumor efficacy of iNKT cells across various malignancies, including gastric cancer and neuroblastoma, where iNKT cell-based therapies have shown potential in improving clinical outcomes and patient survival rates (143, 144). In conclusion, the growing body of evidence indicates that iNKT cell therapy is safe and effective. This highlights the urgent need for more extensive multicenter trials to confirm these findings across various patient demographics and cancer types.

### 2.9 EAL cell therapy

Expanded Activated Lymphocyte (EAL) cell therapy involves extracting and enhancing lymphocytes from a patient’s own body using specialized techniques. These lymphocytes, primarily T cells and natural killer (NK) cells, are then expanded and activated in a laboratory setting before being reinfused into the patient to boost their ability to fight tumors. Both T cells and NK cells play essential roles in immune surveillance (145). Research indicates that EAL cells can recognize tumor-specific antigens and trigger tumor cell death through cytotoxic mechanisms. Additionally, they can modulate the activity of other immune cells by releasing cytokines, thereby creating a robust anti-tumor immune network (146). The proliferation of T and B lymphocytes, along with the presence of cytokines, has shown encouraging results in combating various tumors (147, 148).

One significant study highlighted a Phase I clinical trial that used autologous tumor-infiltrating lymphocytes in patients with primary hepatocellular carcinoma, providing initial evidence for the feasibility of this immunotherapeutic approach in treating this type of cancer (92). Furthermore, a multicenter, randomized, open-label pivotal Phase II study (NCT05213637) evaluated the effectiveness and safety of EAL therapy in preventing recurrence in patients with primary HCC who are at high risk for recurrence after radical resections.

### 2.10 CAR-macrophages cell therapy

Macrophages possess unique characteristics that make them well-suited for CAR (Chimeric Antigen Receptor) engineering. These cells are highly adaptable, allowing them to polarize into different functional states, such as M1 (pro-inflammatory) and M2 (anti-inflammatory) phenotypes. This adaptability enables macrophages to respond to the changing conditions within the tumor microenvironment effectively. As a result, CAR-engineered macrophages (CAR-Ms) can directly attack tumor cells through processes like phagocytosis, while modulating the immune response by influencing the activities of other immune cells (149, 150). CAR-M engineering equips them with remarkable antigen recognition and targeting capabilities. They are specifically designed to identify tumor-associated antigens accurately. For instance, CAR-Ms targeting Glypican-3 (GPC3) are adept at recognizing and eliminating hepatocellular carcinoma (HCC) cells due to their antigen-specific recognition domains on the cell surface (151). Beyond targeting surface antigens, CAR-Ms can also recognize additional markers, such as fibroblast activation protein (FAP), present in the tumor microenvironment. This diverse targeting ability significantly enhances the effectiveness of immune surveillance and the clearance of tumor cells (152). CAR-Ms play a crucial role in the secretion of cytokines and the modulation of the immune response. Research shows that these engineered macrophages produce a range of pro-inflammatory cytokines, such as interferon-gamma (IFN-γ), tumor necrosis factor-alpha (TNF-α), and interleukin-12 (IL-12), which are essential for generating a strong anti-tumor immune response (149, 153). Additionally, CAR-Ms enhance anti-tumor immunity by influencing the activity of other immune cells; for example, they can promote T-cell activation and proliferation through the release of cytokines, leading to a more vigorous immune response (154). Furthermore, CAR-Ms impact the immune environment within the tumor microenvironment by encouraging the polarization of M1 macrophages while suppressing M2 macrophage activity, thereby strengthening anti-tumor effects (155). This inherent ability to modify the tumor microenvironment gives CAR-Ms significant potential for cancer therapy, especially in tackling solid tumors where they can effectively address the immune suppression challenges posed by the tumor microenvironment (156).

Initial findings from clinical trials indicate promising results for using CAR macrophages in treating hepatocellular carcinoma (HCC). One study that combined GPC3-targeted CAR macrophage cells with sorafenib showed significant anti-tumor activity, particularly in smaller tumors, although the effectiveness decreased in larger tumor masses (151). Overall, this combination therapy was more advantageous for smaller tumors, yet it still demonstrated improved anti-tumor properties. Additionally, CAR macrophages targeting CD147 exhibited encouraging anti-tumor effects in laboratory studies, with early evidence hinting at their potential use in clinical applications (157). In preclinical models of HER2+ solid tumors, which generally show low responsiveness to anti-PD1 (aPD1) monotherapy, CAR macrophages with aPD1 proved exceptionally effective. This approach controls tumor growth, extends survival, and modifies the tumor microenvironment (TME). These findings suggest a synergistic relationship between CAR macrophages and T-cell checkpoint inhibitors, highlighting a potential strategy to enhance the response of tumors that usually do not respond to aPD1 therapy in patients (158).

## 3 Challenges and future research directions

### 3.1 Development of new cell therapy technologies

The utilization of CAR-T/NK/M cell therapies in treating hepatocellular carcinoma faces several challenges, particularly regarding target selection due to the tumor’s heterogeneity and the immunosuppressive characteristics of the tumor microenvironment. Future efforts should focus on improving CAR construct designs, which includes identifying more specific and practical tumor-associated antigens and finding ways to mitigate the immunosuppressive effects of the tumor microenvironment. For instance, combining immune checkpoint inhibitors or other immunomodulatory agents could enhance the effectiveness of CAR-T, NK, and M cell therapies. Gene-editing technologies like CRISPR/Cas9 may be utilized to modify T, NK, and M cells, potentially increasing their ability to recognize and target specific antigens found in hepatocellular carcinoma (53, 159, 160). Several studies have attempted to engineer CAR-T, NK, and M cells targeting specific antigens in hepatocellular carcinoma, such as glypican-3 (GPC3), showing promising initial results; however, further improvements and optimizations are necessary (30, 151, 161).

Tumor-infiltrating lymphocytes hold significant potential in cancer immunotherapy, yet their application in hepatocellular carcinoma is still relatively limited. Future research should concentrate on efficiently isolating and expanding TILs from hepatocellular carcinoma tissues and optimizing TIL reinfusion protocols to enhance their therapeutic impact in managing this type of cancer (162). Studies have highlighted the considerable effectiveness of TIL therapy in treating other cancers, like melanoma, providing a helpful reference for its potential use in hepatocellular carcinoma (163).

Mesenchymal stem cells (MSCs) possess both immunomodulatory properties and the ability to regenerate tissues, making them a promising cellular source for treating hepatocellular carcinoma (164). Research has shown that MSCs can exert anti-tumor effects by regulating the tumor microenvironment and promoting apoptosis in cancerous cells. However, further studies are needed to clarify the therapeutic mechanisms through which MSCs function. Additionally, advancements in MSC-based cell therapy products, such as genetic modifications of MSCs or the incorporation of therapeutic agents, could enhance their anti-tumor effectiveness (165).

Exosomes, nanoscale vesicles secreted by cells, can transport specific proteins from their parent cells and serve as alternatives to immune cells by stimulating the production of cytotoxic T lymphocytes, thus contributing to anti-cancer responses (166, 167). More research is required to understand the mechanisms that govern exosome functionality. It is also crucial to optimize exosome preparation and modification techniques to improve their targeting abilities and therapeutic efficacy in managing hepatocellular carcinoma (168, 169). Furthermore, exosomes can be engineered to specifically target and incorporate antigen fragments associated with hepatocellular carcinoma, potentially boosting their anti-cancer properties (170).

The NIR-II laser activates nanomaterials, such as polymer nanoagonists and immunoprotease nanorestimulators, to create localized hyperthermia, reaching temperatures between 45°C and 50°C. This heat directly destroys tumor cells and initiates a process known as immunogenic cell death (ICD). As a result, damage-associated molecular patterns (DAMPs) like ATP, HMGB1, and calreticulin are released, which help mature dendritic cells (DCs) and improve antigen presentation (171). NIR-II photothermal immunotherapy presents a promising and low-toxicity strategy for cancer treatment through four main mechanisms: photothermal ablation, antigen release triggered by ICD, targeted delivery of immunomodulators using activatable nanocarriers, and the combined activation of both innate and adaptive immune responses (172).

### 3.2 Exploration of combination therapy strategies

The integration of cell therapy with immune checkpoint inhibitors represents a promising advancement in the treatment of hepatocellular carcinoma, particularly with the use of PD-1/PD-L1 inhibitors, which have shown significant effectiveness (173). However, there is still a pressing need to improve the response rates seen with monotherapy. By combining cell therapy with immune checkpoint inhibitors, we may achieve synergistic effects; cell therapy can activate the immune system, while immune checkpoint inhibitors can help reduce immunosuppressive mechanisms, ultimately enhancing the anti-tumor immune response (174). Currently, a clinical trial is in progress to assess the effectiveness of CAR-T cell therapy when used alongside PD-1/PD-L1 inhibitors in patients with hepatocellular carcinoma (175). Additionally, **Table 1** provides a comprehensive overview of the mechanisms, benefits, limitations, and potential future directions for various cell and combination therapies.

**Table 1:** Presentation of the mechanisms, advantages, limitations and future development directions of different types of cell therapies and combination therapies.

Cell Therapy Type	Mechanism	Advantages	limitations	Future Research Directions
CAR-T cell therapy	Genetically engineered T cells expressing chimeric antigen receptors (CARs) to target tumor antigens.	High specificity; Potent tumor cell killing	Tumor microenvironment suppression; off-target effects; cytokine release syndrome (CRS).	Develop multi-target CAR-T cells; Combine with immune checkpoint inhibitors; Optimize persistence and activity in the TME.
NK cell therapy	NK cells recognize and kill tumor cells via innate immunity.	High safety profile; no risk of CRS; Adaptable to HCC heterogeneity.	TME suppression; limited *in vivo* expansion and persistence.	Develop CAR-NK with cytokines; Investigate the interaction between NK cells and TME.
TILs cell therapy	Tumor-infiltrating lymphocytes (TILs) are isolated, expanded ex vivo, and reinfused into patients. Naturally tumor-specific, recognize multiple tumor antigens.	Naturally tumor-specific; Recognize various tumor antigens.	Complex isolation and expansion process; TME suppression of TIL function.	Optimize isolation and expansion techniques; Combine with immune checkpoint inhibitors; Identify HCC-specific TIL targets.
Stem cell therapy	Stem cells differentiate into hepatocytes or secrete anti-inflammatory factors to repair liver damage and inhibit tumor growth.	Liver tissue repair; Immunomodulatory effects.	Potential to promote tumor growth; limited therapeutic efficacy.	Investigate stem cell roles in TME; Develop genetically engineered stem cells; Explore combination therapies.
TCR-T cell therapy	Genetically engineered T cells expressing specific T cell receptors (TCRs) to recognize tumor antigens.	Broad target range (including intracellular antigens); Adaptable to HCC heterogeneity.	Risk of autoimmune reactions; complex manufacturing process.	Develop high-efficacy, safe TCR-T cells; Combine with immune checkpoint inhibitors; Optimize target selection.
CIK cell therapy	Heterogeneous CD3+ CD56+ cells with nonspecific cytotoxicity via Fas/FasL and perforin.	Broad antitumor activity (hematologic and solid tumors); Simple preparation and high autologous safety; Mild adverse effects	High interpatient variability in efficacy; Poor *in vivo* proliferation and persistence; Lack of antigen-specific targeting.	Combine with dendritic cells (DC-CIK) to enhance antigen specificity; Engineer CIK cells to express chemokine receptors (e.g., CXCR4) for improved homing; Optimize culture conditions to enrich CD3+ CD56+ subsets.
DC-CIK cell therapy	Dendritic cells (DCs) prime T cells with tumor antigens, while CIK cells mediate MHC-unrestricted killing, synergistically enhancing antitumor immunity.	Dual-action synergy with antigen-specific responses; Applicable to advanced solid tumor; Manageable toxicity.	Complex manufacturing and high costs; Low DC antigen-loading efficiency; Inconsistent clinical outcomes.	Standardize antigen-loading techniques (mRNA electroporation); Combine with ICIs (anti-CTLA-4); Explore cryopreservation to maintain cell viability.
iNKT cell therapy	iNKT cells recognize CD1d-presented glycolipids (α-GalCer), directly killing tumors and activating NK/CD8+ T cells via IFN-γ secretion.	Allogeneic applicability (non-HLA restricted); Immune microenvironment modulation (Treg/MDSC suppression);	Low endogenous iNKT cell frequency in patients; Immature expansion protocols; CD1d expression dependency for antigen presentation.	Develop CAR-iNKT for enhanced targeting; Combine with oncolytic viruses to induce CD1d expression; Optimize ex vivo expansion (IL-7/IL-15).
EAL cell therapy	Anti-CD3 antibody-activated polyclonal T cells mediate tumor killing via perforin/FasL pathways; Enhance immunity via IFN-γ/TNF-α secretion.	Multi-target coverage reducing antigen escape; Mild self-limiting side effects	Mechanism ambiguity and lack of specificity; Efficacy dependent on patient T cell quality;	Isolate high-activity T cell subsets (e.g., CD8+ memory T cells); Combine with chemotherapy to enhance antigen release; Develop cryopreservation protocols for stable cell products.
CAR-M cell therapy	CAR-engineered macrophages phagocytose tumors secrete matrix metalloproteinases (MMPs) to degrade extracellular matrix; activate T cells via MHC-II antigen presentation.	Intense solid tumor infiltration; Reprogramming immunosuppressive microenvironment (M1 polarization); Low CRS risk.	Short *in vivo* persistence (days); Low gene-editing efficiency (macrophage resistance to transduction); Limited targetable surface antigens.	CAR domains fused with phagocytic signals (FcγR); Gene-edited M1 stabilization (C/EBPα overexpression)

Combining Cell Therapy with targeted therapeutics has shown promise in enhancing cancer treatment outcomes. Targeted agents like sorafenib and lenvatinib are effective in suppressing tumor cell growth and forming new blood vessels, but a major challenge is the development of drug resistance over time (176). Combining cell therapy with these targeted treatments may create a synergistic effect by utilizing different mechanisms of action, which could help reduce the chances of resistance (177). For example, studies have found that administering CAR-T cell therapy alongside sorafenib leads to better anti-tumor responses in animal models of liver cancer (178).

On the other hand, traditional treatment methods such as surgery, liver transplantation, radiotherapy, and chemotherapy remain vital in managing hepatocellular carcinoma. By integrating cell therapy with these conventional approaches, we can leverage the benefits of both to improve treatment results (179). For instance, using cell therapy as an additional treatment after surgical removal of tumors or liver transplants can help eliminate any remaining cancer cells and lower the risk of the cancer returning (180). Additionally, combining cell therapy with radiotherapy or chemotherapy may enhance the destructive effects on cancer cells while reducing the side effects commonly associated with these traditional treatments (181, 182).

Integrating various cell therapies showcases unique characteristics and benefits of each approach. When multiple cell therapies are combined, they can create a synergistic effect that significantly improves therapeutic outcomes (183). For example, using CAR-T cell therapy alongside cytokine-induced killer cell therapy or tumor-infiltrating lymphocyte cell therapy allows for the targeted specificity of engineered cells to work in tandem with the broad-spectrum action of natural immune cells, enhancing the management of tumor growth (184). In previous experiments, a construct known as Ad5f35-anti-GPC3-CAR, which utilized a chimeric adenoviral vector (Ad5f35), demonstrated impressive antigen-specific phagocytosis and tumor cytotoxicity (151).

### 3.3 Discovery and application of biomarkers

Alpha-fetoprotein (AFP) is an important biomarker used to diagnose hepatocellular carcinoma (HCC), but some patients with HCC may still test negative for AFP. This highlights the need for alternative biomarkers that provide better sensitivity and specificity. Other potential biomarkers include serum alpha-L-fucosidase (AFU), gamma-glutamyl transpeptidase isoenzyme II (γ-GT2), des-gamma-carboxy prothrombin (DCP), and Golgi protein 73 (GP73), all of which have shown promise in diagnosing and differentiating HCC (185). However, further research is needed to assess their clinical usefulness and the methods for detecting them (186). Additionally, advancements in genomics, transcriptomics, and proteomics may lead to the discovery of new biomarkers such as circulating tumor DNA (ctDNA), microRNA (miRNA), and long non-coding RNA (lncRNA) (187–189). These emerging biomarkers could provide a more accurate basis for the early diagnosis, evaluation of treatment effectiveness, and prediction of outcomes in HCC. By performing gene sequencing and analyzing biomarkers in HCC patients, researchers can gain insights into their tumors’ molecular features and biological behaviors, which can help develop personalized treatment plans. For patients with specific gene mutations or abnormal biomarker levels, targeted therapies or cellular treatments may improve the effectiveness and safety of their care (190). Furthermore, these biomarkers can be used to monitor how healthy treatments are working and to assess the risk of cancer recurrence, allowing for timely adjustments to treatment strategies (191).

The absence of standardized detection methods and criteria for biomarkers presents significant challenges to the accuracy and reliability of their clinical applications (192). To ensure the precision and comparability of future test results, it is crucial to develop uniform protocols for biomarker detection and establish quality control frameworks. Furthermore, conducting multi-center, large-scale clinical studies is essential to validate these biomarkers’ clinical relevance and potential applications.

## 4 Conclusion

Recently, cellular therapies have shown significant promise in managing hepatocellular carcinoma (HCC), leading to better outcomes for patients. This review focuses on the latest advancements in cellular therapies, such as tumor-infiltrating lymphocytes, engineered T cells, and stem cell-based strategies, all of which have yielded encouraging results in preclinical and clinical trials. The treatment landscape for HCC is complex, requiring careful consideration of various factors when incorporating cellular therapies into standard treatment plans. Despite the positive findings, several challenges remain that need further investigation. These challenges include the heterogeneity of HCC, the nature of the tumor microenvironment, and the risk of immune evasion. Moreover, a thorough assessment of the manufacturing processes for cellular products, patient selection criteria, and the long-term safety and effectiveness of these therapies is crucial. It is important to integrate diverse perspectives from various studies to develop a more nuanced understanding of the treatment landscape and avoid overestimating cellular therapies’ effectiveness. Combining cellular therapies with established treatment methods, such as surgery, chemotherapy, and immunotherapy, could create synergistic effects that enhance overall treatment effectiveness. This integrative approach can potentially improve response rates in HCC patients, extend survival, and enhance quality of life. As the field progresses, promoting collaboration among researchers, clinicians, and regulatory bodies will be essential to tackle the complexities of HCC treatment and to ensure the safe and effective use of cellular therapies. In summary, cellular therapies offer promising possibilities for the future management of hepatocellular carcinoma (HCC), but ongoing research and clinical trials are essential to overcome the existing challenges. By fostering a balanced discussion around diverse research findings, we can pave the way for innovative treatment strategies to benefit patients facing this challenging cancer.
